# Supplementary material for: Highly efficient blue organic light-emitting diodes based on carbene-metal-amides
Source: Nat Commun. 2020 Apr 9;11:1758. doi: 10.1038/s41467-020-15369-8 (PMC7145843; doi:10.1038/s41467-020-15369-8)
Supplement: Supplementary file 1 — Supplementary Information [file 41467_2020_15369_MOESM1_ESM.pdf]

Supplementary Information for

Highly Efficient Blue Organic Light-Emitting Diodes Based on Carbene-Metal-Amides

Conaghan et al.

Supplementary Figures

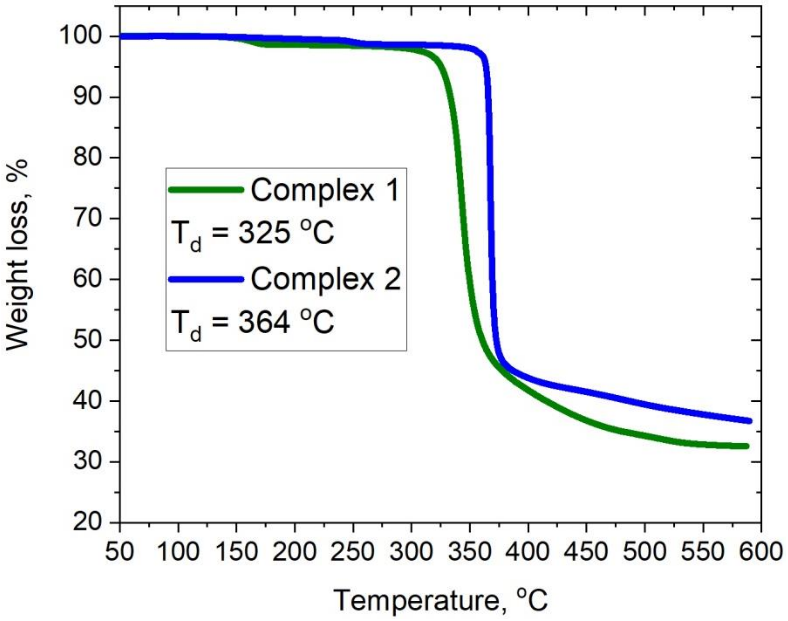

Supplementary Figure 1. TGA curves for gold complexes 1 and 2.

Thermal gravimetric analysis (TGA) for complexes **1** and **2** with a decomposition temperature (T_d).

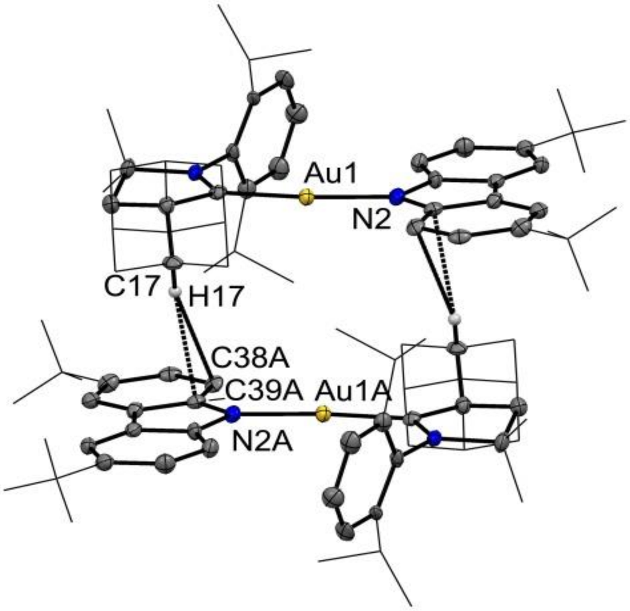

Supplementary Figure 2. Intermolecular interactions for complex 2.

The scheme for the intermolecular C–H···π interaction for complex **2**. Ellipsoids are shown at 50 % probability.

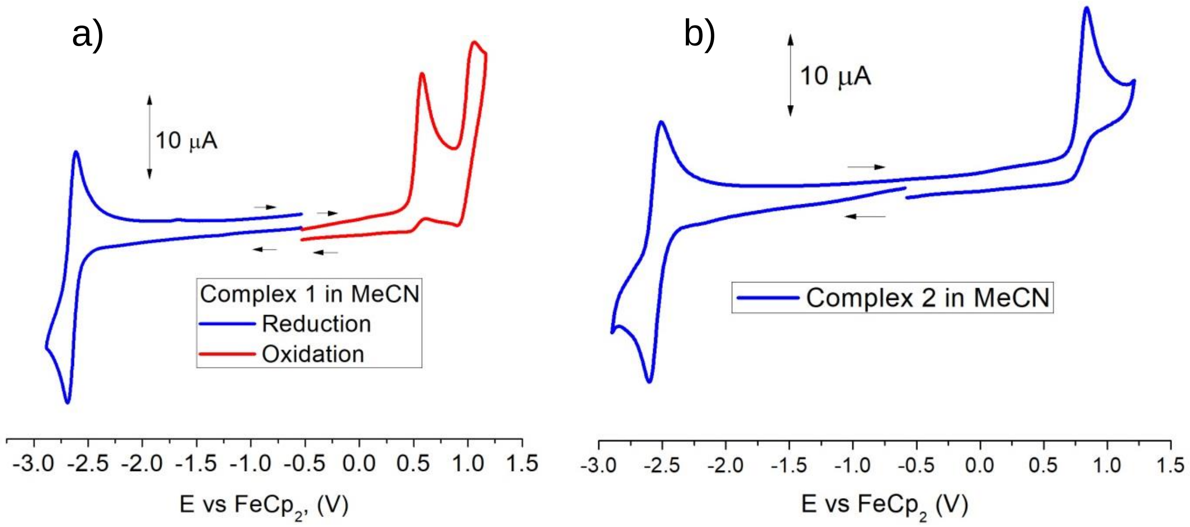

Supplementary Figure 3. Cyclic voltammetry for complexes 1 and 2.

(a) reduction (blue) and oxidation (red) cyclic voltammetry scans for gold complex **1**. (b) Full range cyclic voltammogram for gold complex **2**. Recorded using a glassy carbon electrode in MeCN solution (1.4 mM) with $[n\text{-}Bu_4N]PF_6$ as supporting electrolyte (0.13 M), scan rate 0.1 Vs^{-1}.

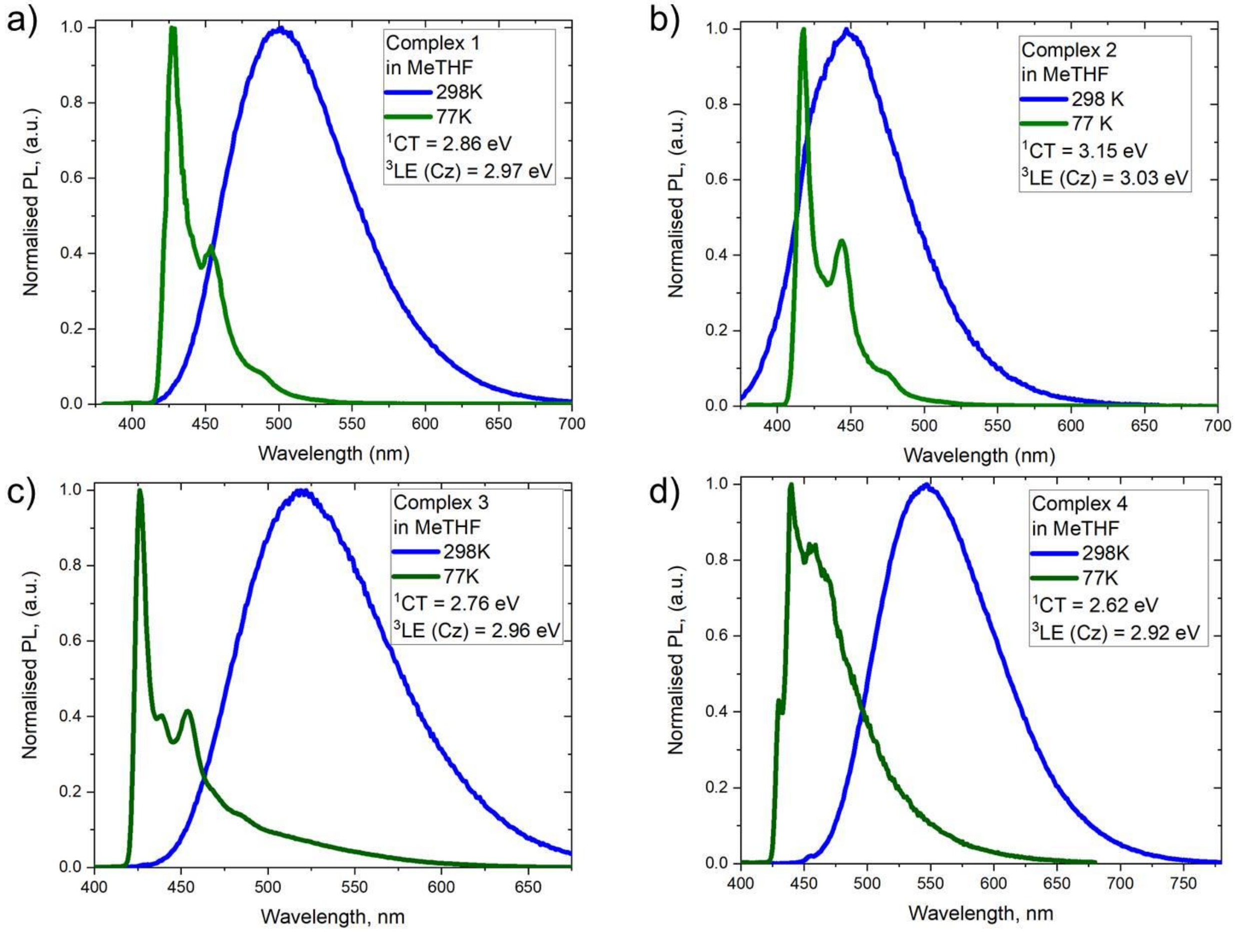

Supplementary Figure 4. Photoluminescence spectra for complex 1–4.

Emission spectra for complexes **CMA1** (a), **CMA4** (b), **1** (c) and **2** (d) in MeTHF solutions at 77 and 298K (excitation at 360 nm, under nitrogen).

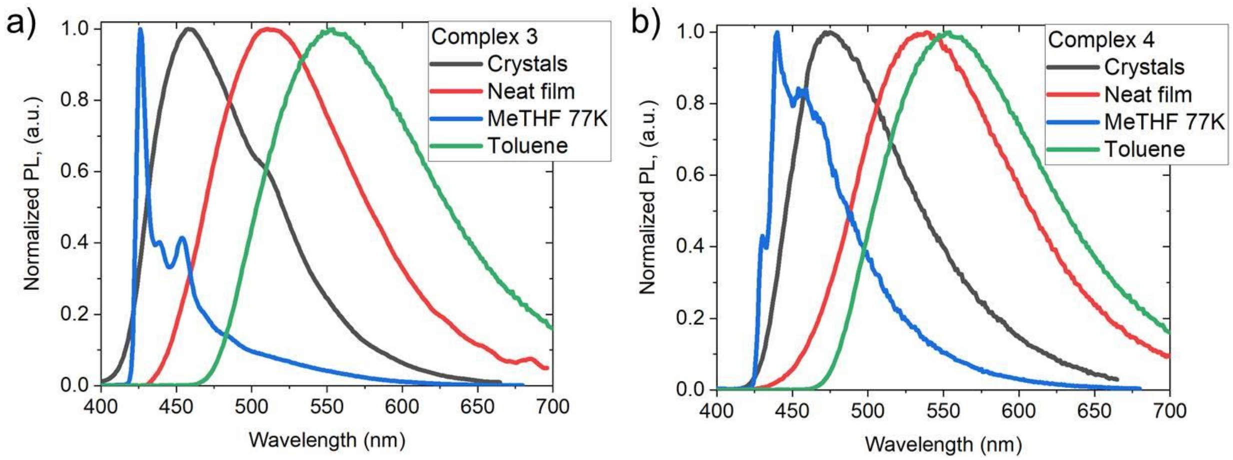

Supplementary Figure 5. Photoluminescence spectra for complex 1–4.

PL spectra of (a) **3** and (b) **4** at 298K as crystals, in neat film, in frozen MeTHF solution and in liquid toluene solution (excitation at 365 nm).

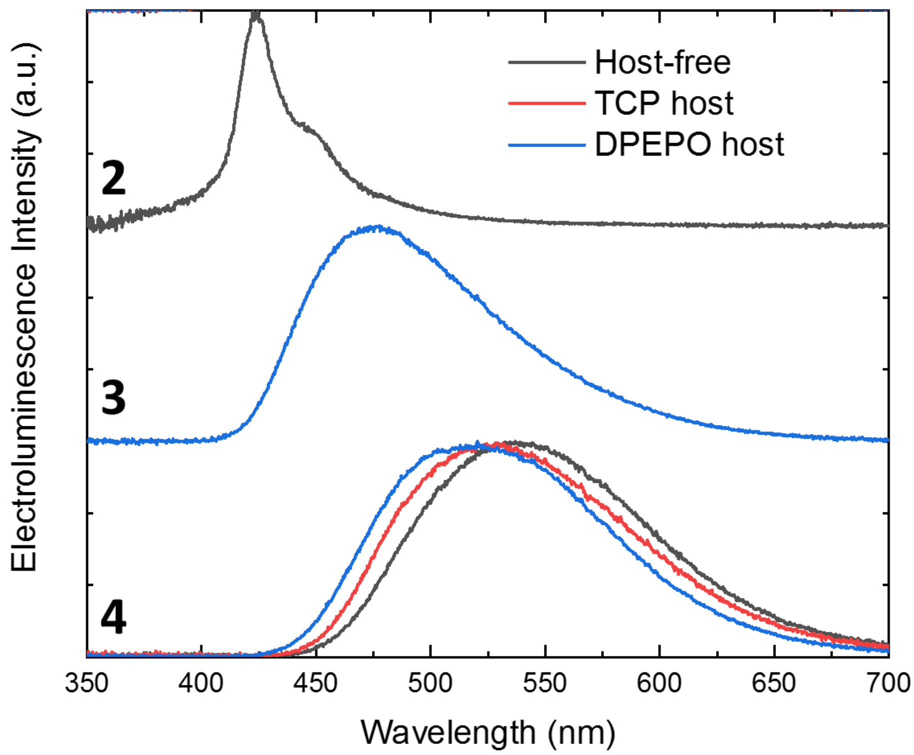

Supplementary Figure 6. Electroluminescence spectra.

Normalised electroluminescence spectra for devices incorporating **2**, **3** and **4** in host-free and host-guest structures.

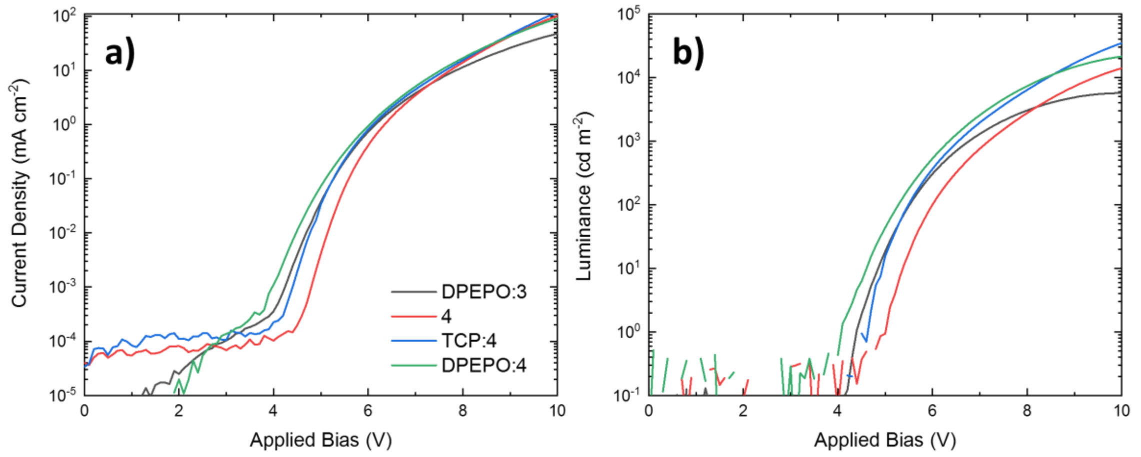

Supplementary Figure 7. Current density-voltage-luminance (J-V-L) curves.

a Current density-voltage. **b** luminance-voltage characteristics for OLEDs based on complexes **3** and **4**

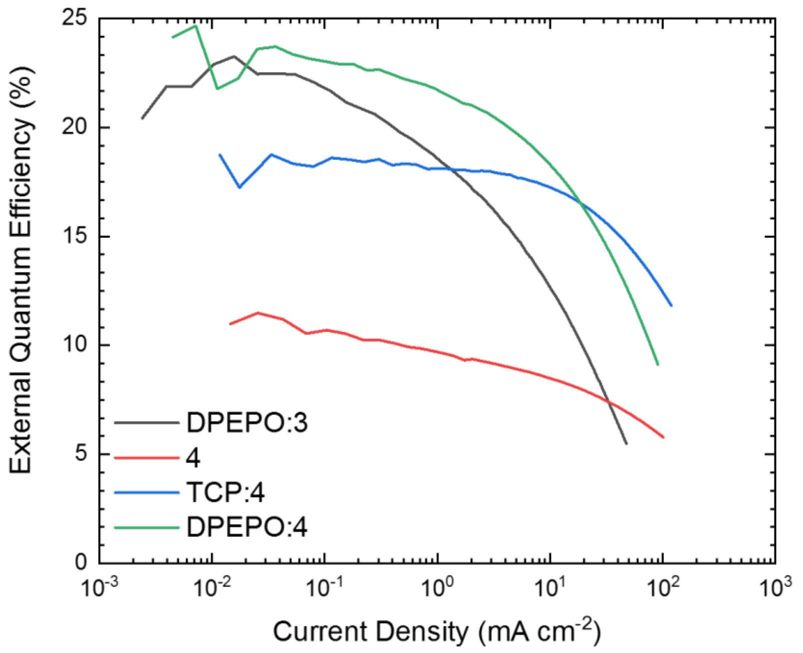

Supplementary Figure 8. External electroluminescence quantum efficiency.

External Quantum efficiency as a function of current density for OLEDs based on **3** and **4**.

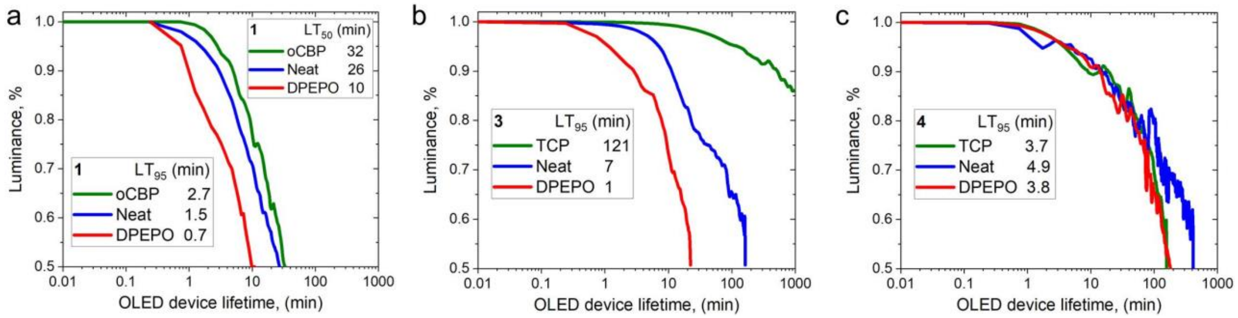

Supplementary Figure 9. OLED device lifetime curves.

OLED device operating lifetime curves (initial luminance 100 cd/m^2) at constant current under vacuum for complexes **1** (a), **3** (b) and **4** (c).

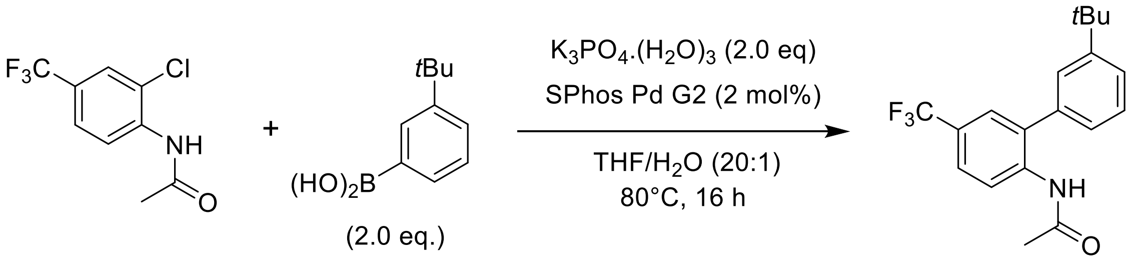

A, 92%

Supplementary Figure 10. Synthesis of compound A.

Synthetic scheme to obtain *N*-(3'-(*tert*-butyl)-5-(trifluoromethyl)-[1,1'-biphenyl]-2-yl)acetamide (**A**).

a

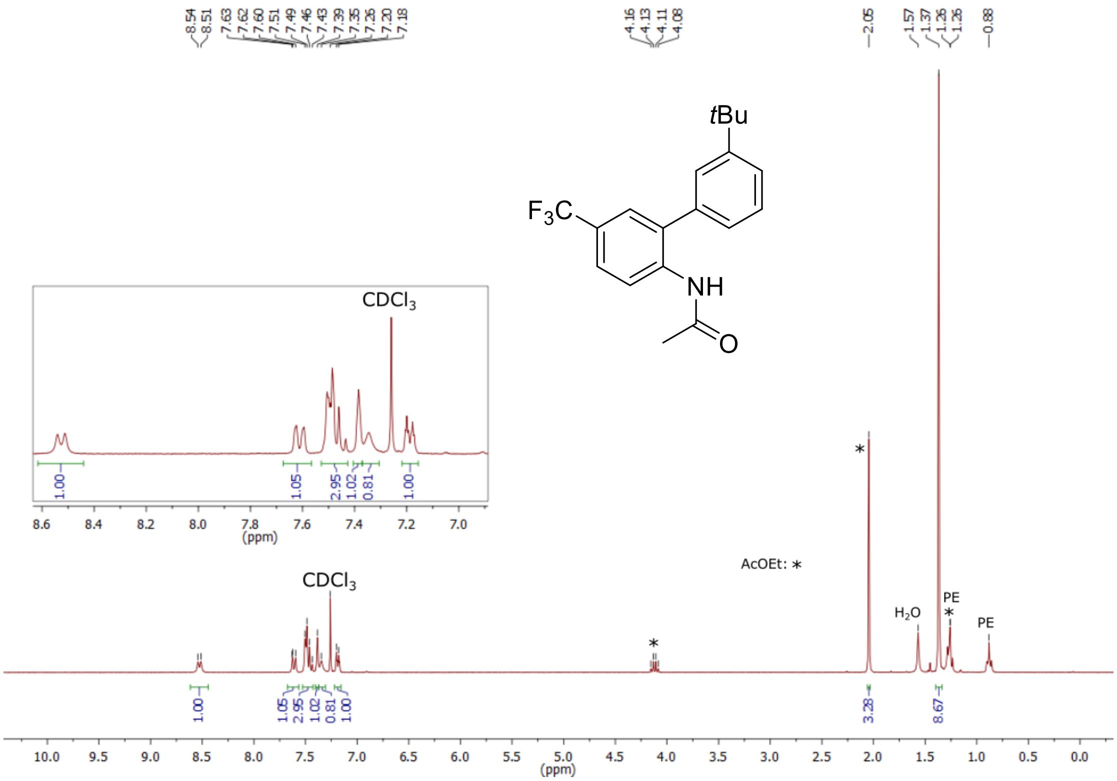
8.54 8.51 7.63 7.62 7.60 7.51 7.49 7.46 7.43 7.39 7.35 7.26 7.20 7.18
4.16 4.13 4.11 4.08
2.05
1.57 1.37 1.26 1.26
0.88
tBu
F_3C
NH
O
$CDCl_3$
1.00 1.05 2.95 1.02 0.81 1.00
8.6 8.4 8.2 8.0 7.8 7.6 7.4 7.2 7.0
(ppm)
AcOEt: *
H_2O
PE
3.28 8.67
10.0 9.5 9.0 8.5 8.0 7.5 7.0 6.5 6.0 5.5 5.0 4.5 4.0 3.5 3.0 2.5 2.0 1.5 1.0 0.5 0.0

b

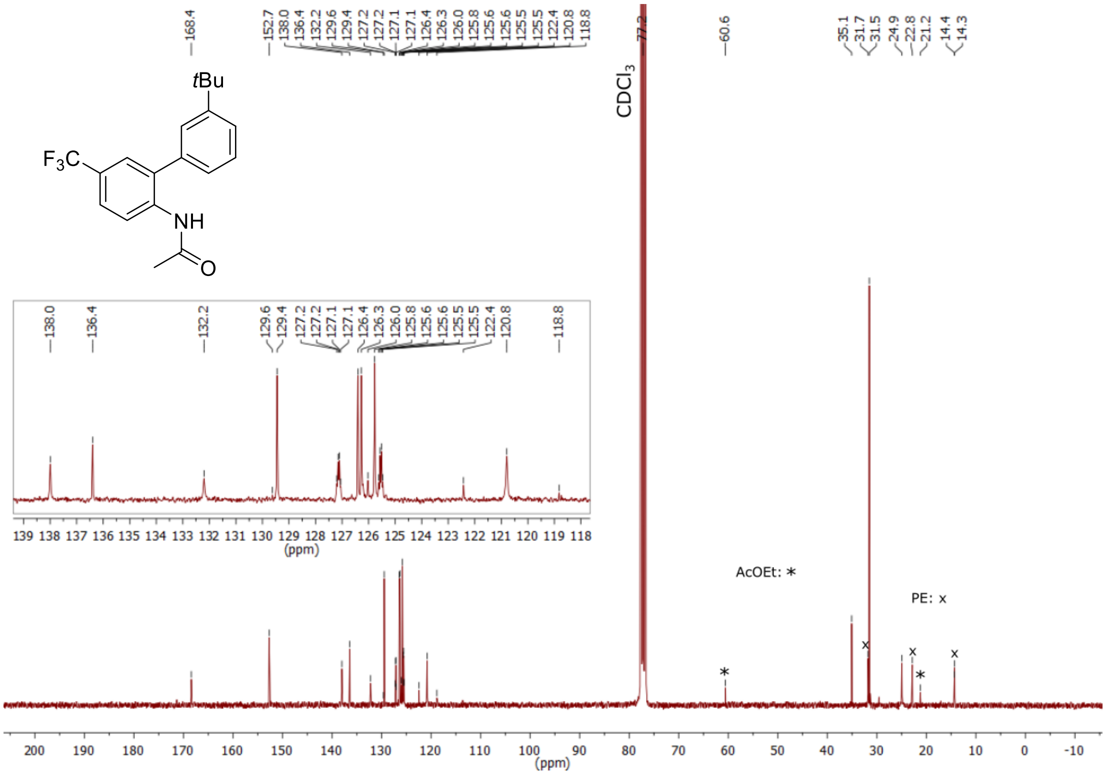
168.4
152.7 138.0 136.4 132.2 129.6 129.4 127.2 127.2 127.1 126.4 126.3 126.0 125.8 125.6 125.5 125.5 122.4 120.8 118.8
77.2
60.6
35.1 31.7 31.5 24.9 22.8 21.2 14.4 14.3
$CDCl_3$
tBu
F_3C
NH
O
139 138 137 136 135 134 133 132 131 130 129 128 127 126 125 124 123 122 121 120 119 118
(ppm)
AcOEt: *
PE: x
200 190 180 170 160 150 140 130 120 110 100 90 80 70 60 50 40 30 20 10 0 -10
(ppm)

c

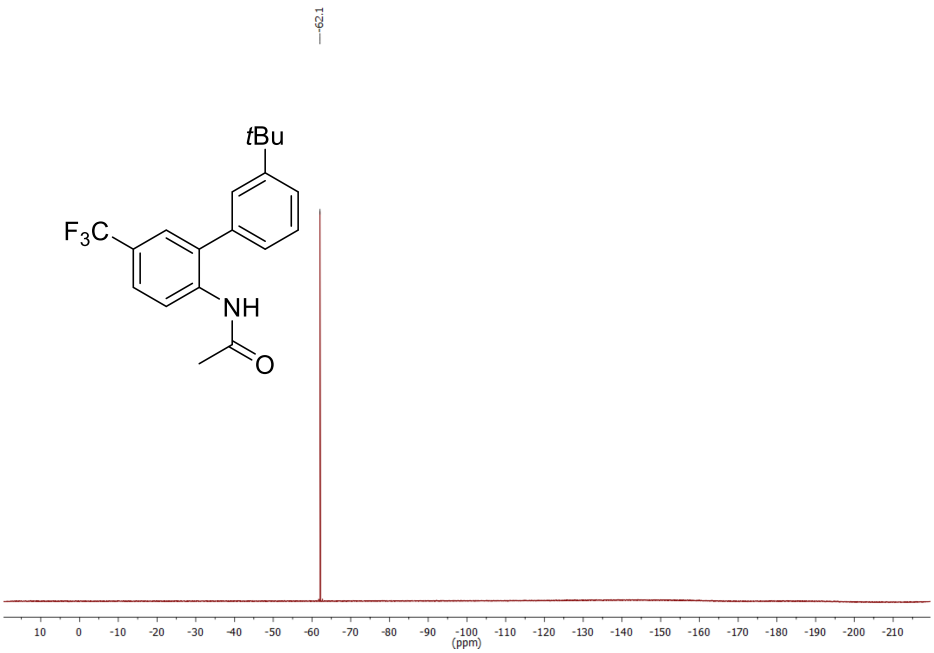

Supplementary Figure 11. NMR spectra for *N*-(3'-(*tert*-butyl)-5-(trifluoromethyl)-[1,1'-biphenyl]-2-yl)acetamide.

(a) ^{1}H NMR (300 MHz, $CDCl_3$); (b) ^{13}C NMR (75 MHz, $CDCl_3$); (c) ^{19}F NMR (282 MHz, $CDCl_3$).

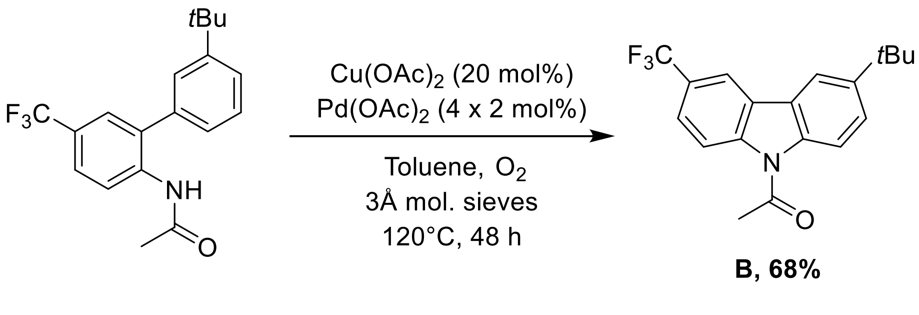

Supplementary Figure 12. Synthesis of compound B.

Synthetic scheme to obtain 6-(*tert*-butyl)-3-(trifluoromethyl)-9-acetylcarbazole (**B**).

a

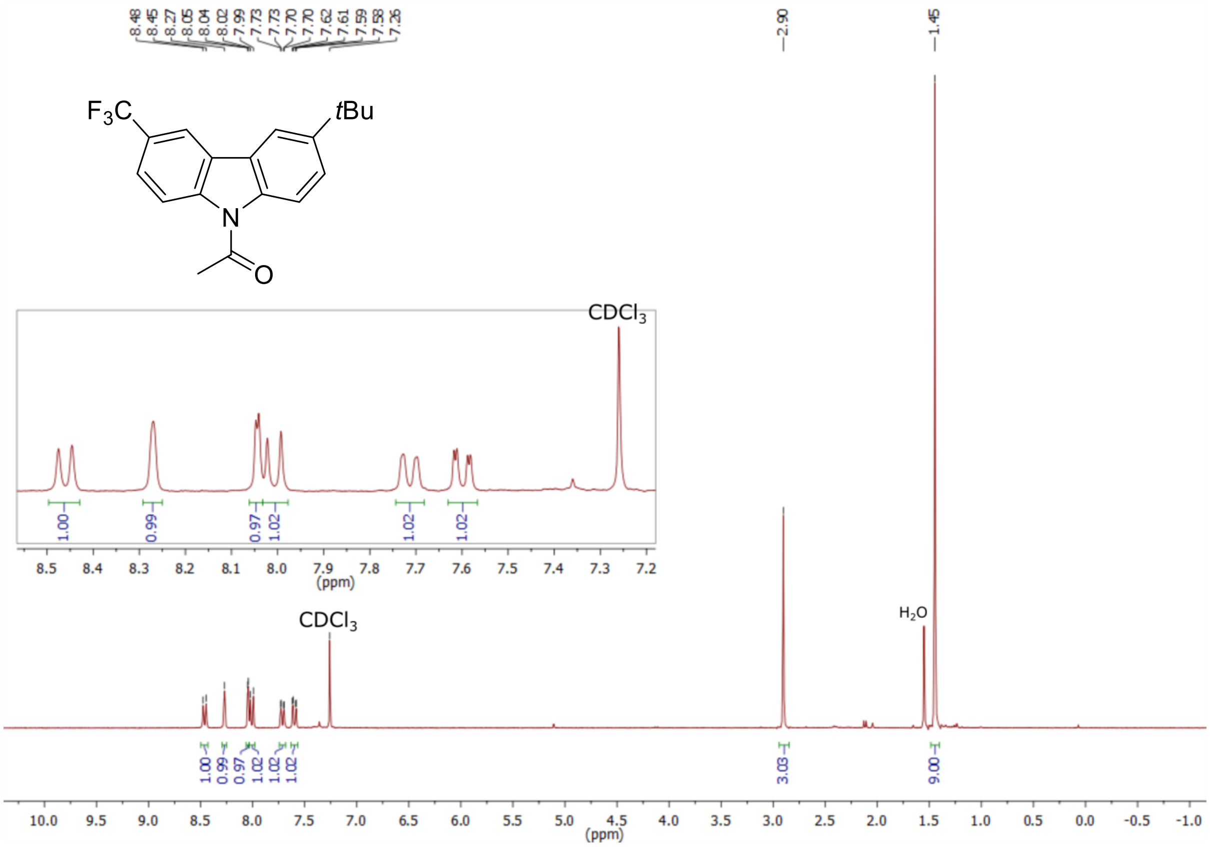

8.48
8.45
8.27
8.05
8.04
8.02
7.99
7.73
7.73
7.70
7.70
7.62
7.61
7.59
7.58
7.26
2.90
1.45
F3C
tBu
N
O
CDCl3
1.00
0.99
0.97
1.02
1.02
1.02
(ppm)
CDCl3
H2O
3.03
9.00
(ppm)

b

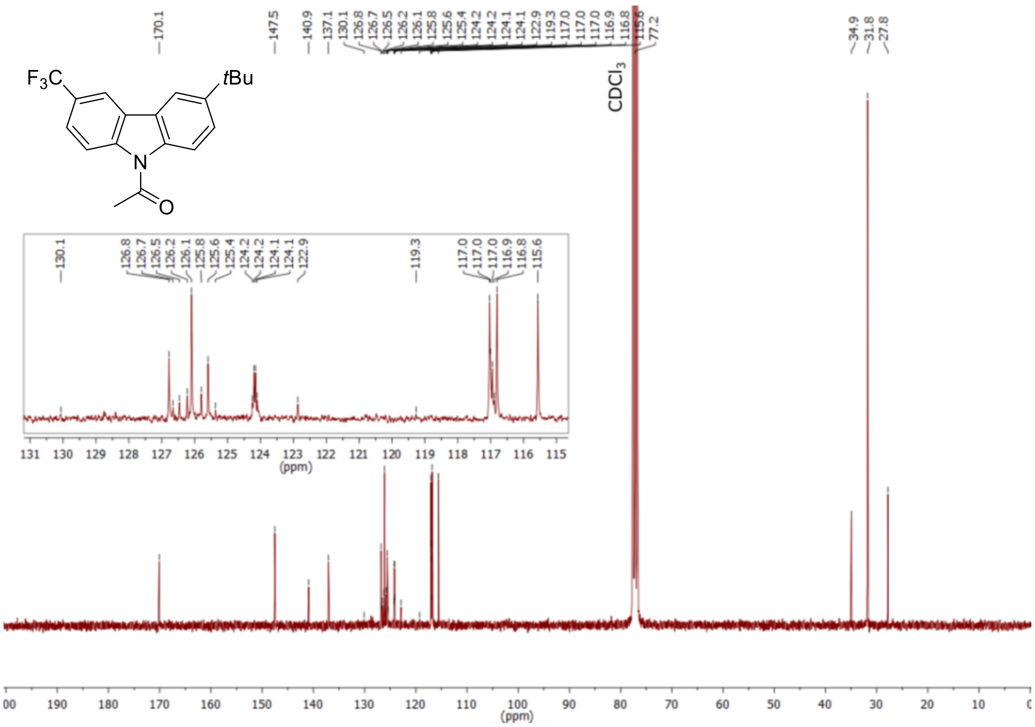

c

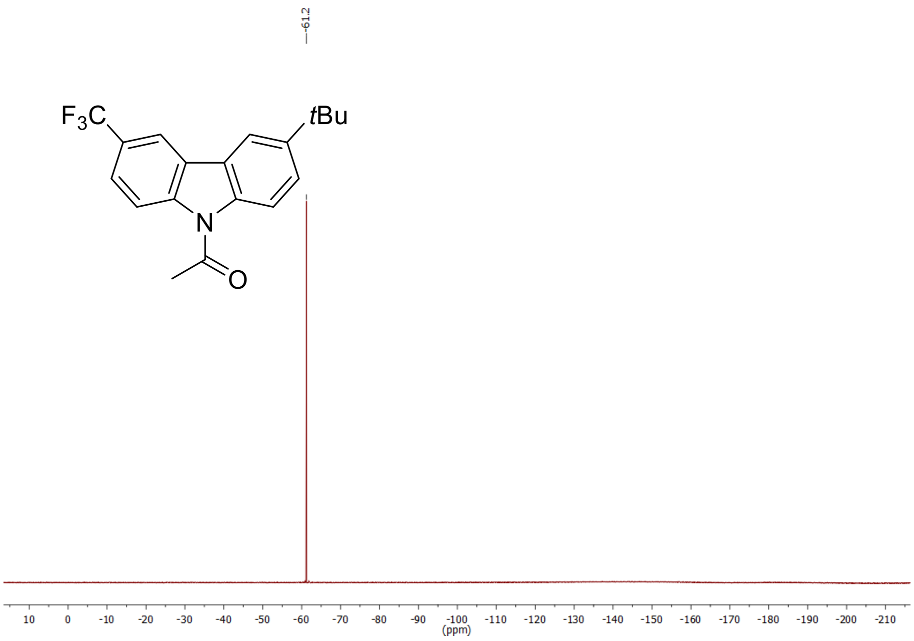

Supplementary Figure 13. NMR spectra for 6-(*tert*-butyl)-3-(trifluoromethyl)-9-acetylcarbazole.

(a) ^{1}H NMR (300 MHz, $CDCl_3$); (b) ^{13}C NMR (75 MHz, $CDCl_3$); (c) ^{19}F NMR (282 MHz, $CDCl_3$).

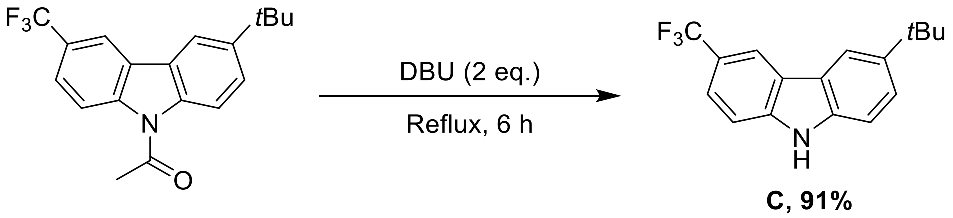

Supplementary Figure 14. Synthesis of compound C.

Synthetic scheme to obtain 6-(*tert*-butyl)-3-(trifluoromethyl)-9H-carbazole (C).

a

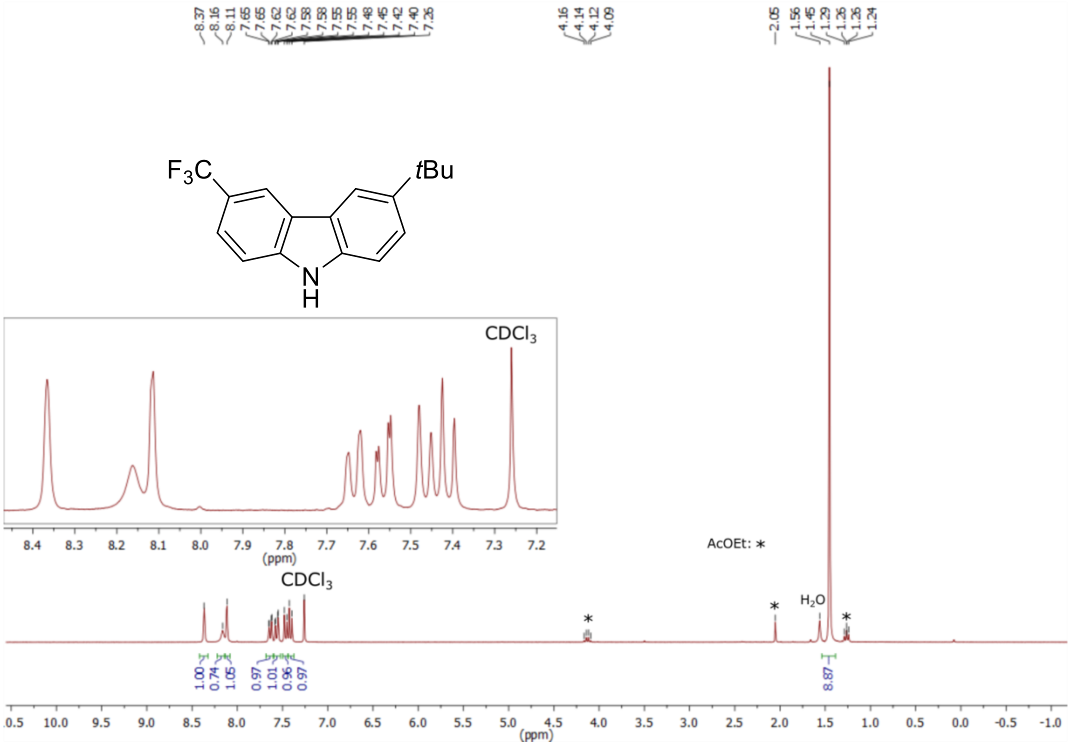

b

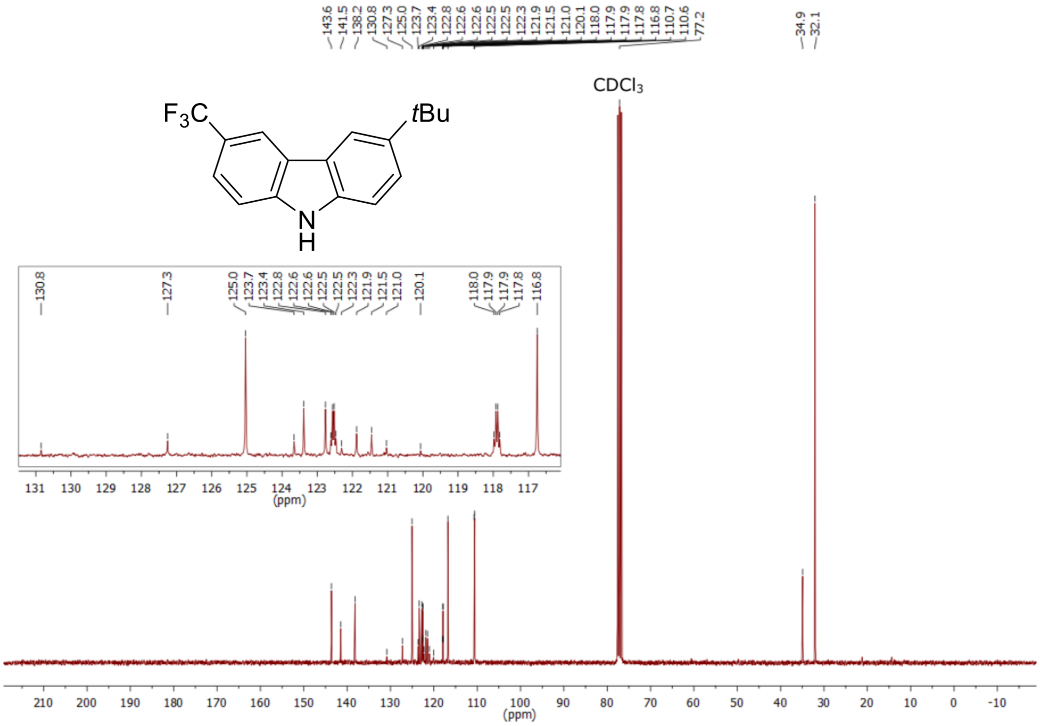

c

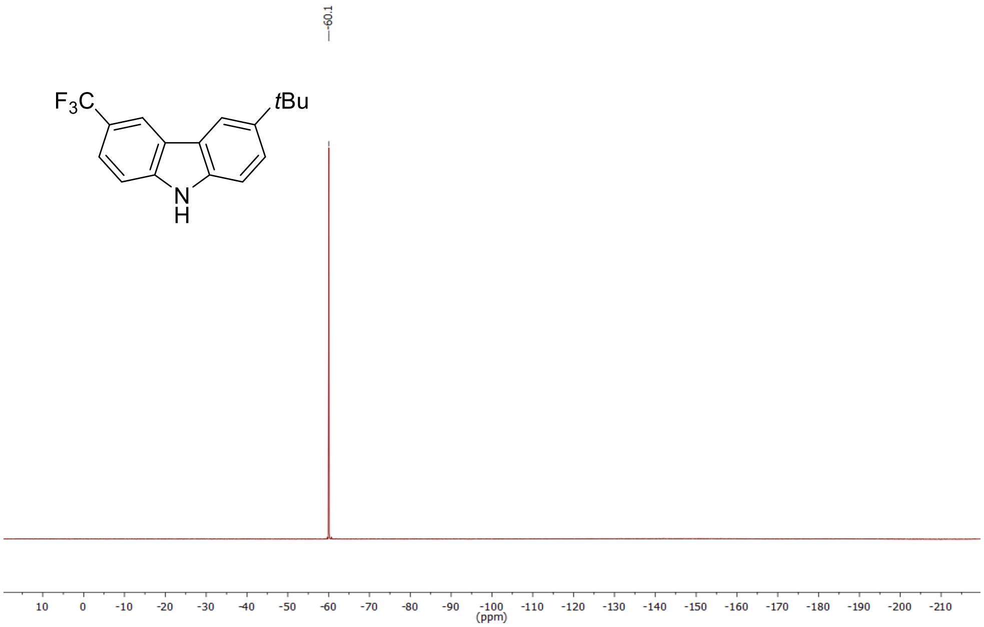

Supplementary Figure 15. NMR spectra for 6-(*tert*-butyl)-3-(trifluoromethyl)-9-acetylcarbazole.

(a) ^{1}H NMR (300 MHz, $CDCl_3$); (b) ^{13}C NMR (75 MHz, $CDCl_3$); (c) ^{19}F NMR (282 MHz, $CDCl_3$).

a

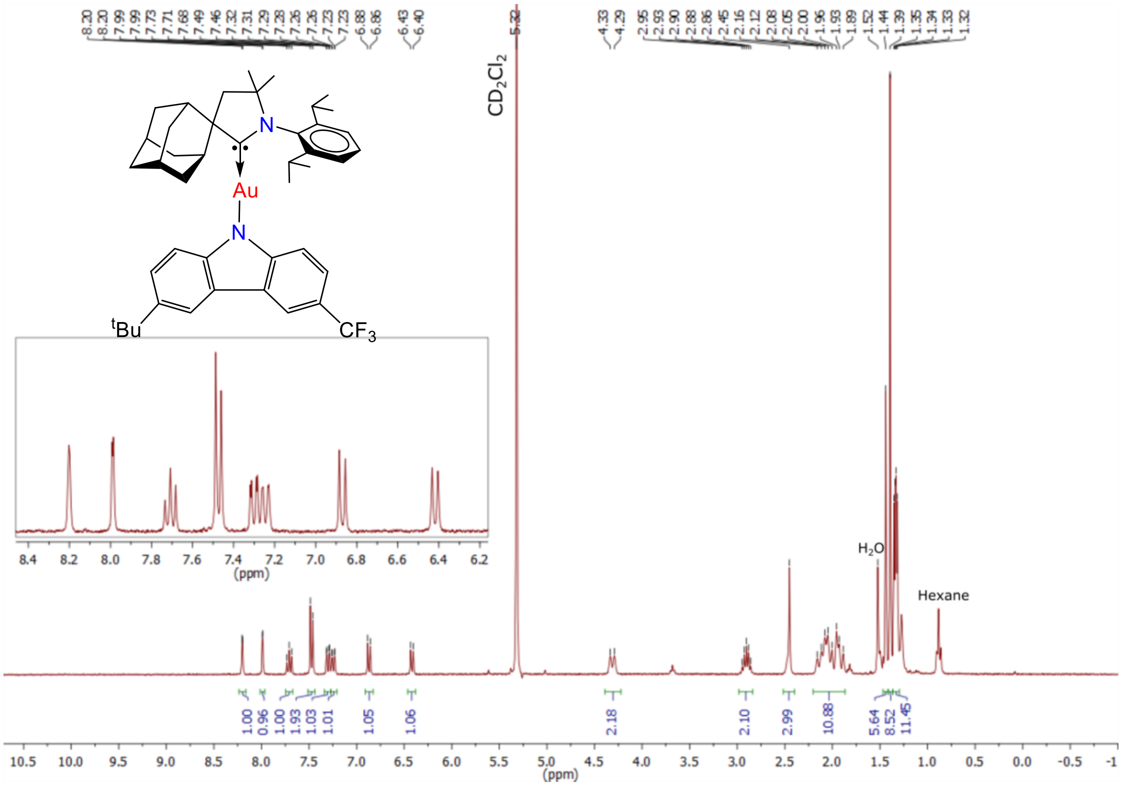
CD2Cl2
Au
N
tBu
CF3
H2O
Hexane
(ppm)

b

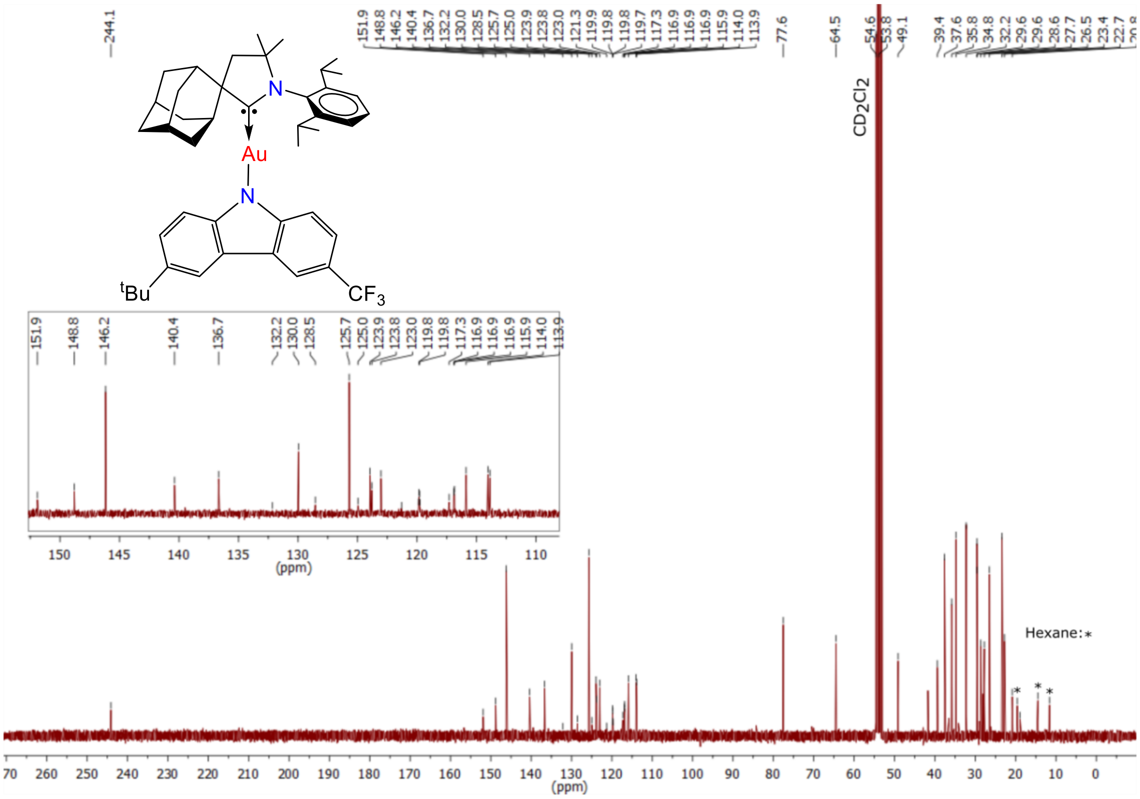
CD2Cl2
Au
N
tBu
CF3
Hexane:*
(ppm)

c

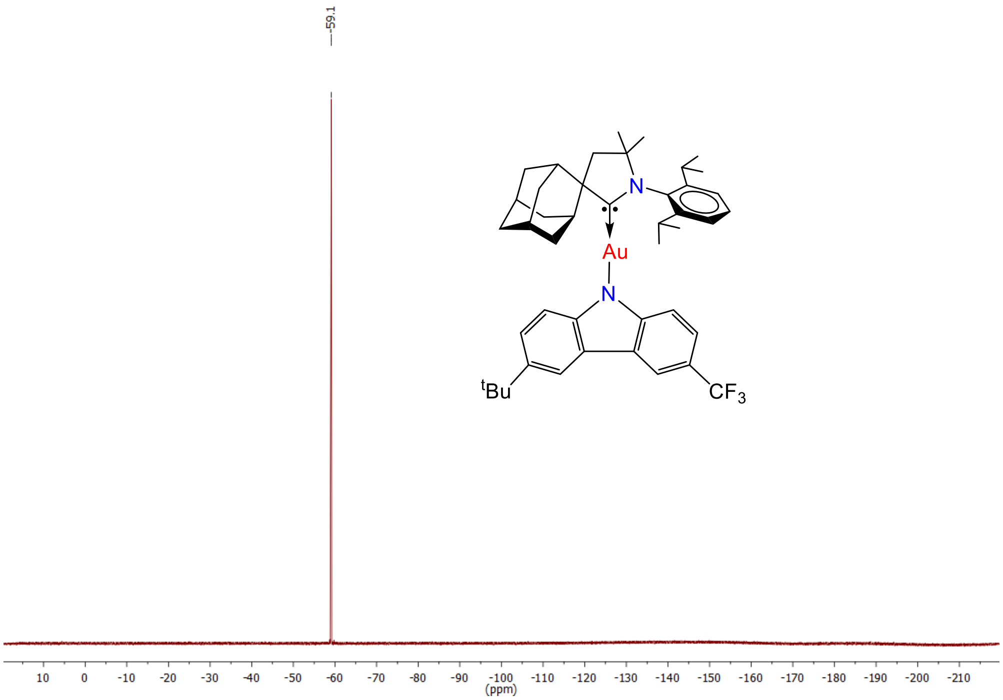

Supplementary Figure 16. NMR spectra for complex 1.

(a) ^{1}H NMR (300 MHz, $CDCl_3$); (b) ^{13}C NMR (75 MHz, $CDCl_3$); (c) ^{19}F NMR (282 MHz, $CDCl_3$).

a

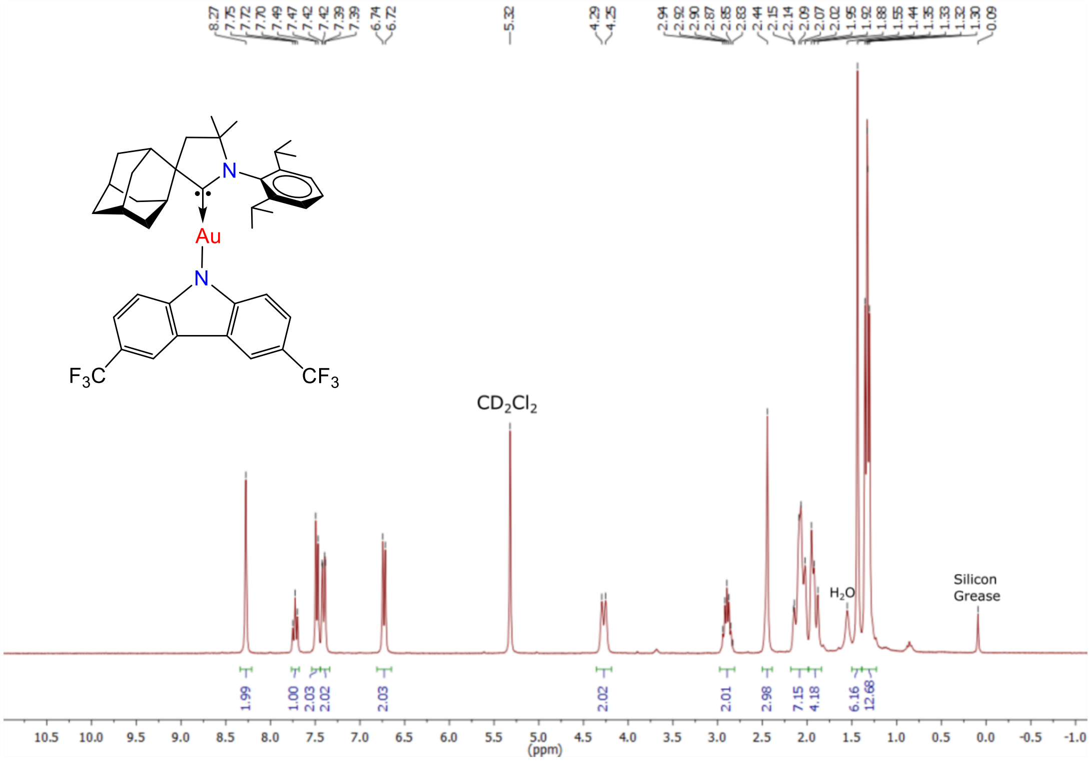
8.27 7.75 7.72 7.70 7.49 7.47 7.42 7.42 7.39 7.39 6.74 6.72
5.32
4.29 4.25
2.94 2.92 2.90 2.87 2.85 2.83 2.44 2.15 2.14 2.09 2.07 2.02 1.95 1.92 1.88 1.55 1.44 1.35 1.33 1.32 1.30 0.09
Au
N
F_3C
CF_3
CD_2Cl_2
H_2O
Silicon Grease
1.99 1.00 2.03 2.02 2.03 2.02 2.01 2.98 7.15 4.18 6.16 12.68
10.5 10.0 9.5 9.0 8.5 8.0 7.5 7.0 6.5 6.0 5.5 5.0 4.5 4.0 3.5 3.0 2.5 2.0 1.5 1.0 0.5 0.0 -0.5 -1.0
(ppm)

b

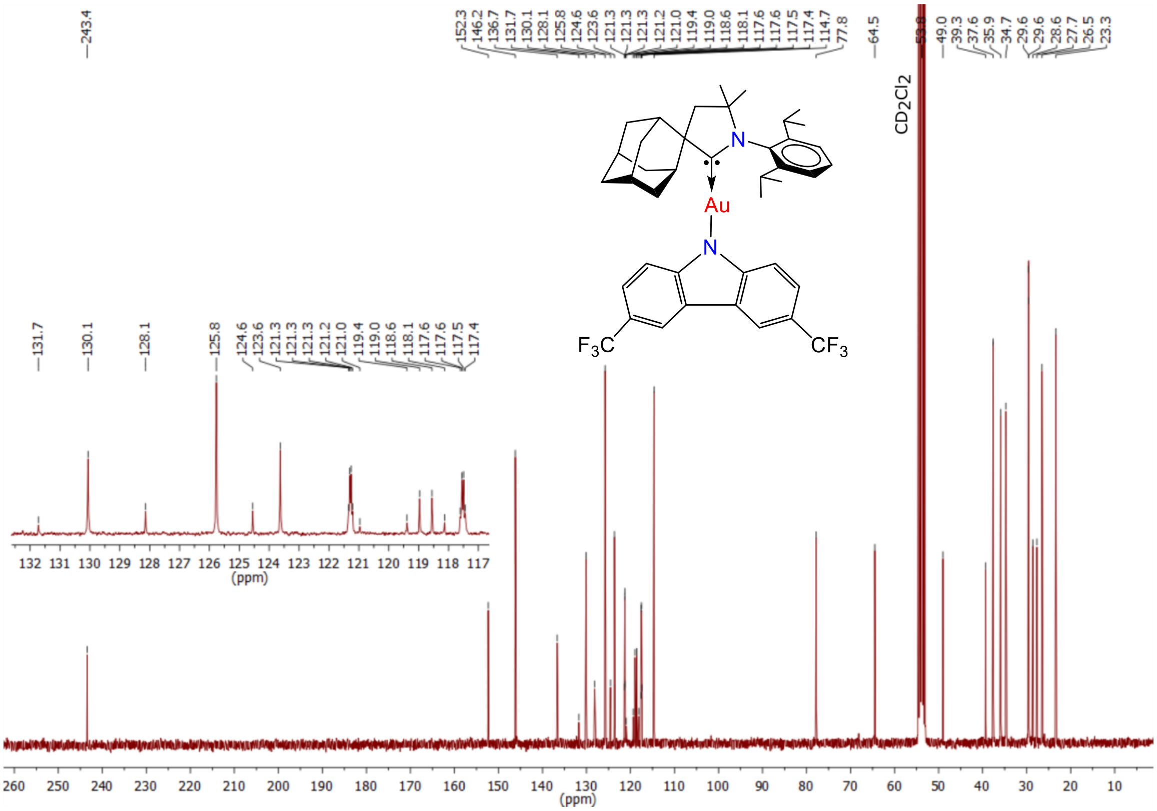
243.4
152.3 146.2 136.7 131.7 130.1 128.1 125.8 124.6 123.6 121.3 121.3 121.3 121.2 121.0 119.4 119.0 118.6 118.1 117.6 117.6 117.5 117.4 114.7 77.8
64.5
53.8
49.0 39.3 37.6 35.9 34.7 29.6 29.6 28.6 27.7 26.5 23.3
CD_2Cl_2
Au
N
F_3C
CF_3
131.7 130.1 128.1 125.8 124.6 123.6 121.3 121.3 121.3 121.2 121.0 119.4 119.0 118.6 118.1 117.6 117.6 117.5 117.4
132 131 130 129 128 127 126 125 124 123 122 121 120 119 118 117
(ppm)
260 250 240 230 220 210 200 190 180 170 160 150 140 130 120 110 100 90 80 70 60 50 40 30 20 10
(ppm)

c

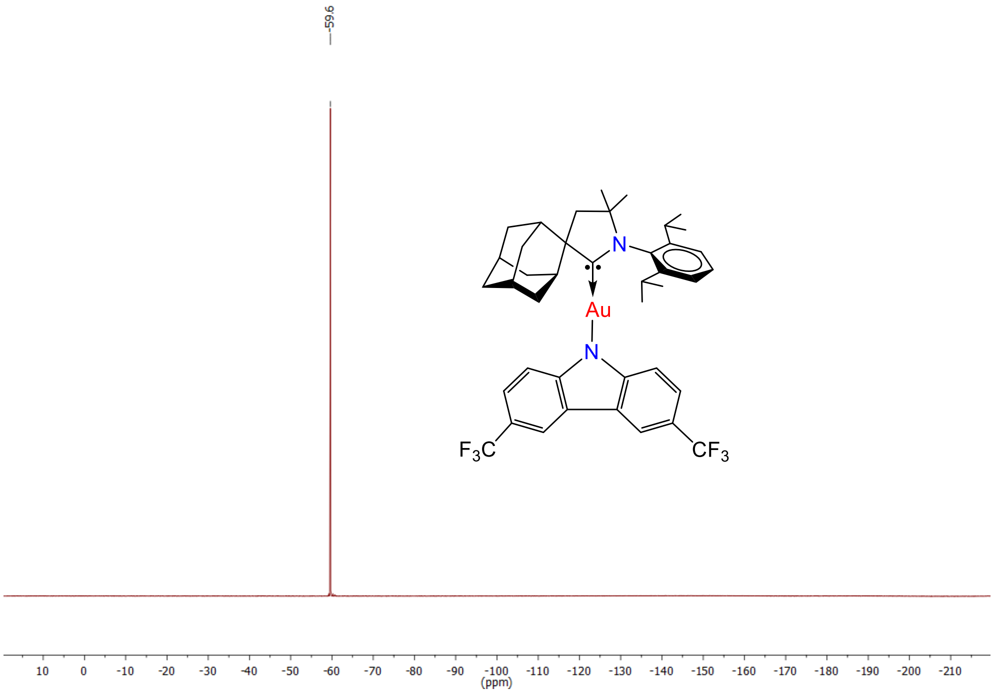

Supplementary Figure 17. NMR spectra for complex 2.

(a) ^{1}H NMR (300 MHz, $CDCl_3$); (b) ^{13}C NMR (75 MHz, $CDCl_3$); (c) ^{19}F NMR (282 MHz, $CDCl_3$).

Supplementary Methods

Synthesis of *N*-(3'-(*tert*-butyl)-5-(trifluoromethyl)-[1,1'-biphenyl]-2-yl)acetamide: *N*-(2-chloro-4-(trifluoromethyl)phenyl)acetamide (1 eq., 5.05 mmol, 1.20 g), 3-(*tert*-butyl)phenylboronic acid (2.0 eq., 10.1 mmol, 1.80 g,) and potassium phosphate trihydrate (2.0 eq., 10.1 mmol, 2.33 g) were mixed in THF/H_2O 20:1 (10 mL) and purged with argon. SPhos Pd G2 (1 mol%, 0.051 mmol, 37 mg) was added and the mixture was heated at 80°C for 16 h. Reaction was cooled to r.t., Et_2O (30 mL) was added and the mixture was filtered through Celite®. The filtrate was diluted with AcOEt (100 mL), washed with water and brine, and dried with $MgSO_4$. The solvent was evaporated and the residue was purified by silica column chromatography (PE/AcOEt) to afford the product as an off-white solid (92%, 1.55 g).

^{1}H NMR (300 MHz, $CDCl_3$): δ 8.53 (d, *J* = 8.7 Hz, 1H), 7.61 (pseudo dd, *J* = 8.7, 2.2 Hz, 1H), 7.52 – 7.42 (m, 3 x 1H overlapped), 7.40 – 7.37 (m, 1H), 7.37 – 7.32 (bs, NH), 7.19 (pseudo dt, *J* = 6.6, 1.9 Hz, 1H), 2.05 (s, 3H, Ac), 1.37 (s, 9H, *t*Bu). ^{13}C NMR (75 MHz, $CDCl_3$) δ 168.4 (s,

C=O), 152.7 (s, C̲–*t*Bu), 138.0 (s, C_q), 136.4 (s, C_q), 132.2 (s, C_q)), 129.5 (s, CH), 127.1 (q, *J* = 3.6 Hz, C̲H–C–CF_3), 126.4 (s, CH), 126.3 (s, CH), 125.8 (s, CH), 125.5 (q, *J* = 3.7 Hz, C̲H–C–CF_3), 124.2 (q, *J* = 271.8 Hz, CF_3), 120.8 (s, CH), 35.1 (s, C̲(CH_3)$_3$), 31.5 (s, C(C̲H_3)$_3$), 25.0 (s, CH_3 Ac), ($\underline{C}_{ipso}$–CF_3 was not observed due to overlap with aromatic signals). ^{19}F NMR (282 MHz, $CDCl_3$) δ -62.1 ppm. Anal. Calcd. for $C_{19}H_{20}F_3NO$ 335.37): C, 68.05; H, 6.01; N, 4.18. Found: C, 68.17; H, 6.18; N, 4.31. See Supplementary Figs. 10 and 11.

Synthesis of 6-(*tert*-butyl)-3-(trifluoromethyl)-9-acetylcarbazole: In an oven-dried Schlenk tube, under argon, *N*-(3'-(*tert*-butyl)-5-(trifluoromethyl)-[1,1'-biphenyl]-2-yl)acetamide (1 eq., 4.47 mmol, 1.50 g), $Cu(OAc)_2$ (20 mol%, 0.89 mmol, 162 mg), $Pd(OAc)_2$ (2 mol%, 0.089 mmol, 20 mg) and 3 Å molecular sieves were mixed in toluene (20 mL). The flask was purged by oxygen and heated at 120°C. The $Pd(OAc)_2$ was added every 8 h (4 × 20 mg). The reaction was monitored by ^{19}F NMR indicating full completion after 48 h. The mixture was cooled to r.t., diluted with AcOEt (60 mL) and filtered through Celite®. The filtrate was diluted with AcOEt (100 mL) washed with water and brine and dried with $MgSO_4$. The solvent was evaporated and the residue was purified by silica column chromatography (PE/AcOEt) to afford the product as an off-white solid (68%, 1.01 g).

^{1}H NMR (300 MHz, $CDCl_3$): δ 8.46 (d, *J* = 8.8 Hz, 1H, CH^1), 8.27 (pseudo s, 1H, CH^4), 8.04 (d, *J* = 2.1 Hz, 1H, CH^5), 8.01 (d, *J* = 8.9 Hz, 1H, CH^8), 7.71 (pseudo dd, *J* = 8.8, 1.9 Hz, 1H, CH^2), 7.60 (dd, *J* = 8.9, 2.1 Hz, 1H, CH^7), 2.90 (s, 3H, Ac), 1.45 (s, 9H, *t*Bu). ^{13}C NMR (75 MHz, $CDCl_3$) δ 170.1 (s, C=O), 147.5 (C̲–*t*Bu), 140.9 (s, C_q), 137.1 (s, C_q), 126.8 (s, C_q), 126.1 (s, CH^7), 126.0 (q, *J* = 32.6 Hz, C̲–CF_3), 125.6 (s, C_q), 124.7 (q, *J* = 271.7 Hz, CF_3), 124.2 (q, *J* = 3.6 Hz, CH^2), 117.0 (s, CH^1 overlapped with CH^4), 116.9 (q, *J* = 3.9 Hz, CH^4 overlapped with CH^1), 116.8 (s, CH^5), 115.6 (s, CH^8), 34.9 (s, C̲(CH_3)$_3$, 31.8 (C(C̲H_3)$_3$, 27.8 (s, CH_3 Ac). ^{19}F NMR (282 MHz, $CDCl_3$) δ -61.2. Anal. Calcd. for $C_{19}H_{18}F_3NO$ (333.35): C, 68.46; H, 5.44; N, 4.20. Found: C, 68.13; H, 5.68; N, 3.97. See Supplementary Figs. 12 and 13.

Synthesis of 6-(*tert*-butyl)-3-(trifluoromethyl)-9H-carbazole. DBU (2 eq., 6.06 mmol, 904 µL) was added to 6-(*tert*-butyl)-3-(trifluoromethyl)-9-acetylcarbazole (1 eq., 3.03 mmol, 1.01 g) in MeOH (30 mL), and the mixture was refluxed for 6 h. The reaction was cooled to r.t. and volatiles were evaporated. AcOEt (120 mL) was added, washed with water and brine, and dried

over $MgSO_4$. The residue was purified by silica column chromatography (PE/AcOEt) to afford the product as a white solid (91%, 800 mg).

^{1}H NMR (300 MHz, $CDCl_3$): δ 8.37 (s, 1H, CH^4), 8.16 (bs, 1H, NH), 8.12 (pseudo s, 1H, CH^5), 7.64 (pseudo dd, J = 8.5, 1.7 Hz, 1H, CH^2), 7.56 (dd, J = 8.6, 1.9 Hz, 1H, CH^8), 7.47 (d, J = 8.5 Hz, 1H, CH^1), 7.41 (d, J = 8.6 Hz, 1H, CH^7), 1.45 (s, 9H, *t*Bu). ^{13}C NMR (75 MHz, $CDCl_3$) δ 143.6 (s, C–*t*Bu), 141.5 (s, C_q), 138.2 (s, C_q), 125.5 (q, J = 271.2 Hz, CF_3), 125.0 (s, CH^7), 123.4 (s, C_q), 122.8 (s, C_q), 122.5 (q, J = 3.7 Hz, CH^2), 121.7 (q, J = 32.1 Hz, C–CF_3), 117.9 (q, J = 4.1 Hz, CH^4), 116.8 (s, CH^5), 110.7 (s, CH^1), 110.6 (s, CH^8), 34.9 (s, $C(CH_3)_3$), 32.1 (s, $C(CH_3)_3$). ^{19}F NMR (282 MHz, $CDCl_3$) δ -60.1. Anal. Calcd. for $C_{17}H_{16}F_3N$ (291.32): C, 70.09; H, 5.54; N, 4.81. Found: C, 69.82; H, 5.72; N, 4.63. See Supplementary Figs. 14 and 15.

X-Ray Crystallography.

Crystals of **1** and **2** suitable for X-ray diffraction study were obtained by layering a toluene solution with hexanes. Complex **1** and **2** crystallizes with two independent molecules in the unit cell and molecules of toluene. The CF_3-group was disordered over two half-populated positions for the complex **1** (independent molecule A). For the final refinement, the contribution of severely disordered toluene molecules in the crystals of **1** was removed from the diffraction data with PLATON/SQUEEZE.[1,2] Crystals were mounted in oil on glass fiber and fixed on the diffractometer in a cold nitrogen stream. Data were collected using an Oxford Diffraction Xcalibur-3/Sapphire3-CCD diffractometer with graphite monochromated Mo K_α radiation (λ = 0.71073 Å) at 140 K. Data were processed using the CrystAlisPro-CCD and –RED software.[3] The structure was solved by direct methods and refined by the full-matrix least-squares against F^2 in an anisotropic (for non-hydrogen atoms) approximation. All hydrogen atom positions were refined in isotropic approximation in a "riding" model with the $U_{iso}(H)$ parameters equal to 1.2 $U_{eq}(C_i)$, for methyl groups equal to 1.5 $U_{eq}(C_{ii})$, where $U(C_i)$ and $U(C_{ii})$ are respectively the equivalent thermal parameters of the carbon atoms to which the corresponding H atoms are bonded. All calculations were performed using the SHELXTL software.[4]

The principal crystallographic data and refinement parameters:

Complex **1**, CCDC number 1916996, $C_{51}H_{62}AuF_3N_2$, Monoclinic, space group $P2_1/n$, a = 23.0603(9) Å, b = 15.7169(5) Å, c = 26.0909(9) Å, β = 103.114(4)°, V = 9209.7(6) Å^3, Z = 8, d_{calc} = 1.380 g cm^{-3}, μ = 3.242 mm^{-1}, colorless/block, crystal size 0.25 × 0.21 × 0.09 mm, F(000)

= 3904, T_{min}/T_{max} 0.73420/1.00000, R_1 = 0.0341 (from 18085 unique reflections with $I>2\sigma(I)$; R_{int} = 0.0449, R_{sigma} = 0.0392) and wR_2 = 0.0852 (from all 73935 unique reflections), *GOF* = 1.096, $\Delta\rho_{min}/\Delta\rho_{max}$ = 1.65/–1.28.

Complex **2**, CCDC number 1916995, $C_{41}H_{45}AuF_6N_2$, Triclinic, space group *P-1*, *a* = 13.2995(6) Å, *b* = 14.8662(6) Å, *c* = 26.4244(10) Å, α = 74.133(4)°, β = 77.531(3)°, γ = 71.883(4)°, *V* = 4727.0(4) Å^3, *Z* = 4, d_{calc} = 1.232 g cm^{-3}, μ = 3.160 mm^{-1}, colorless/block, crystal size 0.16 × 0.11 × 0.07 mm, *F*(000) = 1752, T_{min}/T_{max} 0.70618/1.00000, R_1 = 0.0396 (from 18562 unique reflections with $I>2\sigma(I)$; R_{int} = 0.0548, R_{sigma} = 0.0852) and wR_2 = 0.0754 (from all 41002 unique reflections), *GOF* = 1.022, $\Delta\rho_{min}/\Delta\rho_{max}$ = 1.15/–1.28.

Supplementary References

1. Spek, A. L. Structure validation in chemical crystallography. *Acta Cryst.* **D65**, 148–155 (2009).

2. van der Sluis, P. & Spek, A. L. BYPASS: an effective method for the refinement of crystal structures containing disordered solvent regions. *Acta Cryst.* **A46**, 194–201 (1990).

3. *Programs CrysAlisPro*, Oxford Diffraction Ltd., Abingdon, UK (2010).

4. Sheldrick, G.M. SHELX-97 and SHELX-2018/3 – Programs for crystal structure determination (SHELXS) and refinement (SHELXL), *Acta Cryst.* **C71**, 3–8 (2015).
